# Robot-Assisted Extraperitoneal Ventral Hernia Repair—Experience From the First 160 Consecutive Operations With Lateral eTEP and eTAR Techniques

**DOI:** 10.3389/jaws.2024.13055

**Published:** 2024-11-22

**Authors:** Robert Vogel, Frank Heinzelmann, Peter Büchler, Björn Mück

**Affiliations:** Klinik für Allgemein-, Viszeral- und Kinderchirurgie—Klinikum Kempten, Kempten, Germany

**Keywords:** eTEP, robotic abdominal wall repair, eTAR, retromuscular mesh repair, extraperitoneal mesh placement

## Abstract

**Introduction:**

There is a growing consensus on the benefits of retro-muscular (RM) mesh positioning, highlighted by its recommendation in the latest edition of EHS guidelines. The eTEP method has facilitated minimally invasive hernia repairs with retro-muscular mesh placement. With the increasing availability of robotic systems, there has been a corresponding increase in robotic adaptations of minimally invasive techniques involving retro-muscular mesh placement.

**Materials and Methods:**

All patients who underwent robotic ventral hernia repair using the lateral extraperitoneal eTEP technique at Kempten Hospital between September 2019 and December 2023 were includes in the study. Preoperative characteristics, perioperative parameters, postoperative parameters, and hernia-specific parameters, were retrospectively analyzed using the hospital information system.

**Results:**

160 patients were operated using a lateral approach eTEP technique during the observation period, 111 (69.38%) for incisional hernia repair and 49 (30.63%) for primary hernia repair. 43 cases required TAR (30 unilateral TAR and 13 bilateral TAR). 139 patients had a medial (86.98%), seven patients (4.14%) a lateral and 14 patients (8.88%) a combined hernia defect. The median operative time was 143 min (range: 53 min–495 min). The median length of hospital stay was 3 days (range: 2–16). There was one intraoperative complication. The postoperative complication rate was 6.25% (10 patients), with 1.72% (2 patients) requiring reoperation. Sonographic follow-up examinations revealed seromas in 5 patients, with 4 located in the retromuscular mesh space and 1 in the former hernia sac. None of these seromas required surgical intervention.

**Conclusion:**

The “lateral approach” of robotic eTEP provides a safe surgical method for treating ventral hernias using minimally invasive techniques and mesh augmentation in the retro-muscular space. Further studies are necessary to compare extraperitoneal with transperitoneal methods.

## Introduction

There is increasing consensus regarding the advantages of retro-muscular (RM) mesh positioning, to the point that it has been recommended in the latest edition of guidelines (EHS Guidelines 2023) [1]. In recent years, the conventional approach to minimally invasive hernia repair has been predominated by procedures involving intraperitoneal mesh placement. However, the latest registry analyses conducted across several European nations reveal a relevant decline in the adoption of this method [2, 3]. Concurrently, there has been a notable increase in the development of innovative minimally invasive methods for retro-muscular mesh implantation. This shift is largely driven by the demonstrated benefits of positioning mesh within the retro-muscular space, as outlined in numerous meta-analyses [4–6].

Belyansky et al. [7] first introduced the enhanced-view totally extraperitoneal (eTEP) method as a technique for minimally invasive hernia repair, which includes the placement of mesh in the retro-muscular space. Similarly, the endoscopic mini or less open sublay hybrid technique, published by Schwarz et al. [8] and the laparoscopic transperitoneal sublay mesh repair published by Schroeder [9], have emerged as elegant and promising approaches combining the benefits of minimally invasive surgery with the proven efficacy of retro-muscular mesh positioning.

Unfortunately, these surgical procedures turned out to be technically demanding when performed in a traditional endoscopic or laparoscopic setting. Particularly at the start of the learning curve, they have proven to be very time-consuming, which has led to limited adoption. As a result, these techniques have been primarily performed in select specialized centers.

Alongside the increasing availability of robotic systems, there has been a rise in robotic adaptations of minimally invasive techniques invloving retromuscular mesh placement. These robotic approaches have helped overcome the technical challenges, such as reduced dexterity and precision, often encountered with conventional “straight-stick” laparoscopy. Notable among these adaptations is the transabdominal retromuscular umbilical prosthetic hernia repair (TARUP) technique, introduced by Muysoms et al. [10], and the totally extraperitoneal robotic eTEP (r-eTEP) method outlined by Belyansky et al [11].

Since 2019, our hospital has been applying a robotic system in the treatment of hernias [12]. Robotic operative techniques quickly became our standard approach for ventral hernia repair when mesh implantation was indicated. Within 2 years, the proportion of minimally invasive hernia repairs had risen to 87.5%, with nearly 95% of these procedures achieving extraperitoneal mesh placement.

During the initial phases of the learning curve, transperitoneal procedures such as ventral TAPP and TARUP were used. However, already within the first year, the extraperitoneal eTEP approach for retrorectus repair has become the predominant method for treating ventral hernias. This technique has since become the standard procedure for retromuscular mesh placement, even for more complex cases requiring bilateral TAR.

This study aims to demonstrate the safety and feasability of the extraperitoneal eTEP technique with lateral trocar placement in ventral hernia repair through a retrospective analysis of perioperative outcomes.

## Materials and Methods

All patients who underwent a robot-assisted eTEP procedure between September 2019 and December 2023 at Kempten Hospital were retrospectively analyzed using data retrieved from the hospital’s information system.

During this period, with a few exceptions, nearly all patients indicated for retromuscular or retrorectus mesh placement underwent surgery via the eTEP technique. Exceptions where open surgery was required included emergency cases, hernias with a defect diameter of more than 15 cm, and cases involving a loss of domain. Patients undergoing simultaneous abdominal wall reconstruction were also treated using the open technique. The reasons for opting for a transperitoneal approach during robotic retromuscular repair were either simultaneous intraperitoneal procedures or a recurrence situation where a retromuscular mesh obstructed access to the retrorectus space. Patients with a rectus muscle diameter of less than 5 cm were operated on using the eTEP technique with caudal trocar positioning, as the retrorectus space was too narrow for lateral trocar placement. The IPOM technique was only applied in rare cases, usually in older, multimorbid patients.

All procedures were performed by two surgeons who had prior experience with endoscopic eTEP repair before the introduction of robotic surgery. Both surgeons had exclusive access to the robot for hernia surgery, therefore their learning curves were included in the study.

The following parameters were retrospectively examined: preoperative characteristics (gender, age, ASA status, height, weight, body mass index [BMI]), perioperative parameters (operative time, mesh size, number of meshes, intraoperative complications), postoperative parameters (complication rate, reoperation rate, length of hospital stay), and hernia-specific parameters (type of hernia, hernia size, hernia location). Perioperatively, all patients received antibiotic prophylaxis and postoperatively received thrombosis prophylaxis. Hernia findings were classified according to the European Hernia Society (EHS) classification [13]. Mesh position was designated based on Parker’s classification [14]. The complexity of the procedures was categorized according to the criteria outlined in the publication by Slater et al. [15]. Written consent to participate in the Herniamed quality assurance study was obtained from all patients [16].

### Operative Technique

**FIGURE 1:**
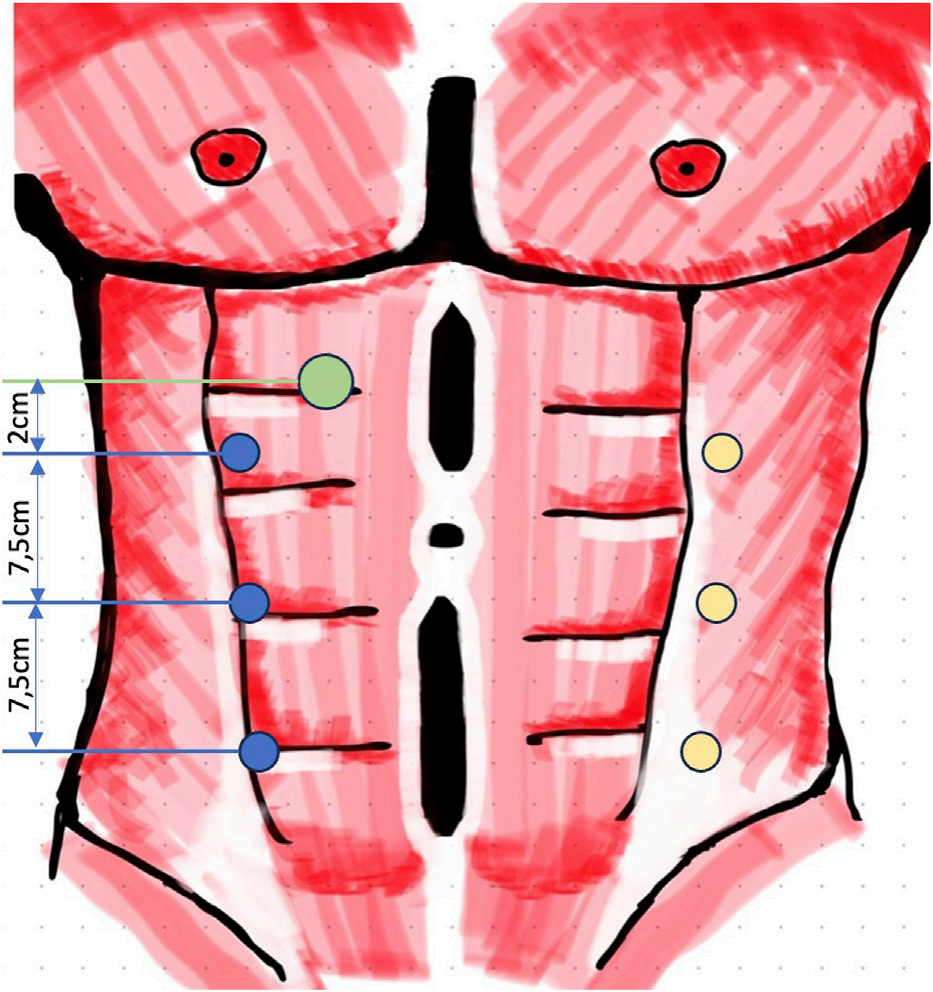
Trocar positioning lateral r-eTEP (green, 12 mm access port; blue, 8 mm robotic ports; yellow, second portline for bilateral TAR with double docking).

All procedures were performed using the DaVinci-X system (Intuitive Surgical, Sunnyvale, CA, United States). The “lateral approach” is described as follows: patients are positioned in a slightly extended supine posture. The side of access is determined based on the width of the rectus sheath, presence of scars, and any additional hernia findings (e.g., inguinal or lateral hernias). The robotic patient cart is placed on the opposite side of the selected access. If double docking is not required, the patient’s arm on the robot-facing side is extended, while the other arm remains aligned with the body. Double docking was only necessary in cases of bilateral transversus abdominis release (TAR) or simultaneous bilateral inguinal hernia repairs. Preoperatively, anatomical landmarks, such as the rib cage, pelvic bones, rectus sheath boundaries, and hernia or scar locations, are marked. In the upper abdomen, a 12-mm optical port is used to access the retrorectus space (Figure 1), with the robotic 8-mm camera already in use for this entry. No additional laparoscopic system is necessary. Balloon dissection can be avoided, as blunt dissection with the camera suffices to create space for the placement of the first 8-mm DaVinci port in the mid-abdomen, medial to the lateral border of the rectus sheath.

**FIGURE 2:**
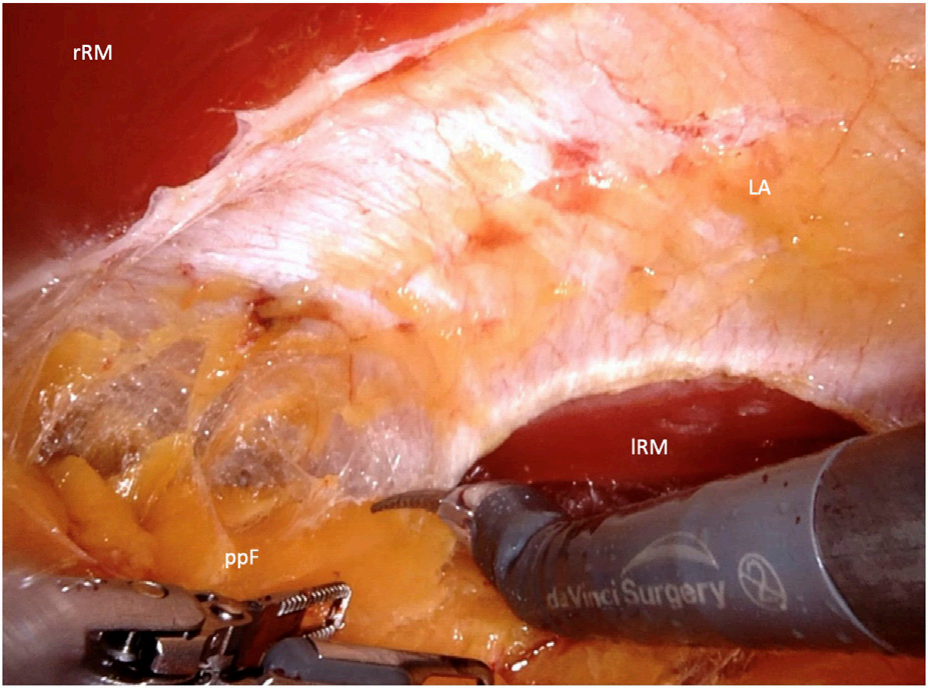
Crossover through the midline in the upper abdomen (rRM, right rectus muscle; lRM, left rectus muscle; LA, linea alba; ppF, preperitoneal fatty tissue).

**FIGURE 3:**
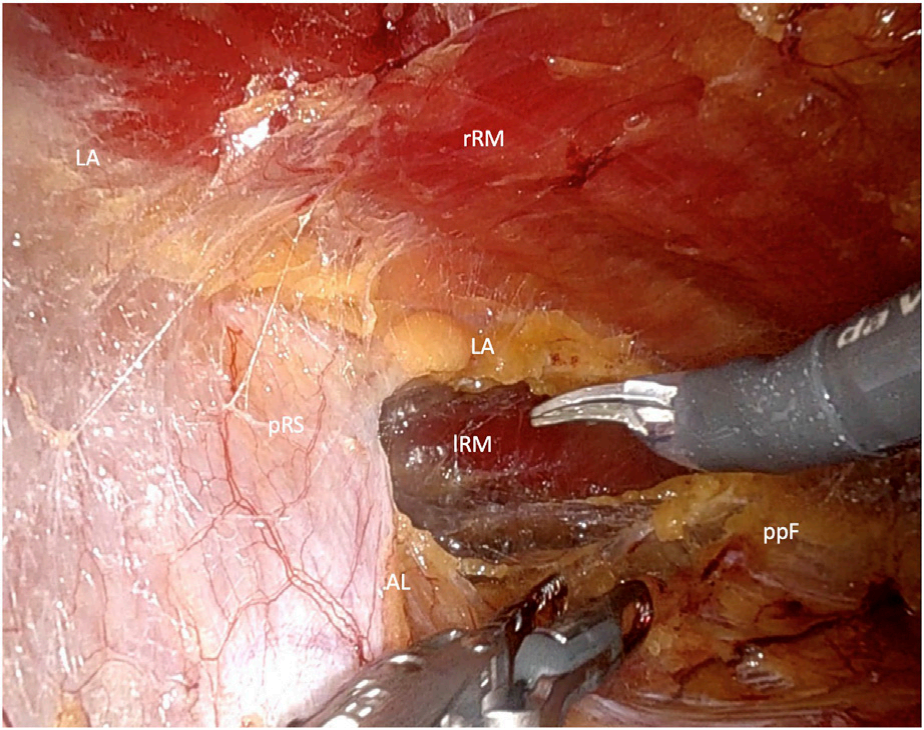
Crossover through the midline in the lower Abdomen (rRM, right rectus muscle; lRM, left rectus muscle; pRS, posterior rectus sheath; LA, linea alba; AL, arquate line; ppF, preperitoneal fatty tissue).

After placement of the first 8-mm DaVinci port, endoscopic dissection of the ipsilateral retrorectus space is continued. Two additional 8-mm DaVinci ports are then inserted along the same line in the upper and lower abdomen. The DaVinci X system is subsequently docked from the opposite side of the patient. The crossover maneuver into the contralateral retrorectus space can be initiated either in the upper abdomen (Figure 2) or the lower abdomen (Figure 3).

In the upper abdomen, a longitudinal incision is made in the posterior rectus sheath, slightly lateral to its transition into the linea alba. This exposes the preperitoneal fat of the falciform ligament, which is carefully dissected from the linea alba. Dissection is carried out to the opposite side, creating a space between the linea alba and preperitoneal adipose tissue unil the contralateral rectus muscle is visualized through the posterior rectus sheath. A similar longitudinal incision is then made on the contralateral side, lateral to the linea alba.

**FIGURE 4:**
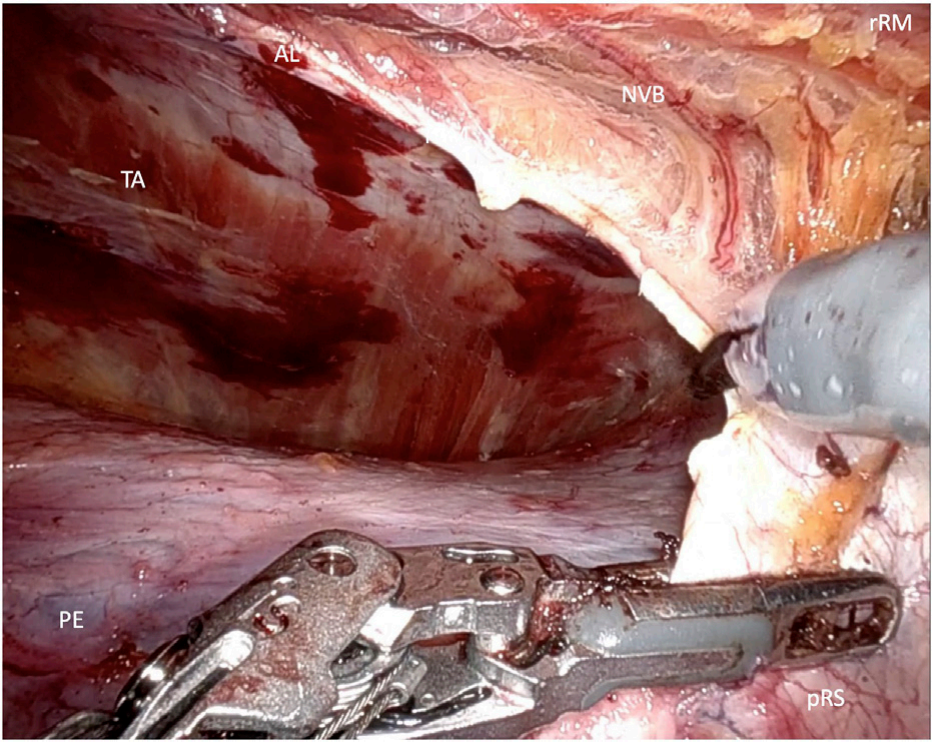
Transversus abdominis release (rRM, right rectus muscle; pRS posterior rectus sheath; AL, arquate line; NVB, neuovascular bundle).

In the lower abdomen, below the arcuate line, where the posterior rectus sheath is absent, the crossover maneuver is completed by dividing the connective tissue of the linea alba that separates the retrorectus spaces on both sides. During this midline dissection, the hernia is mobilized and repositioned. The dissection of the contralateral retrorectus space continues up to the lateral border of the rectus sheath, while preserving neurovascular bundles under direct visualization. If required, a unilateral single docking or bilateral double docking transversus abdominis release (TAR) may be performed (Figure 4).

Any peritoneal perforations are meticulously closed to prevent contact between mesh and the intestine. The anterior rectus sheath is reconstructed with absorbable barbed sutures, and rectus diastasis correction, if necessary, is done concurrently. The dissected space is then measured, and a large, uncoated synthetic mesh is introduced through the optical trocar, ensuring it overlaps all trocar sites. A drainage tube is placed in the retrorectus space, and an abdominal binder is applied to the patient at the conclusion of the procedure.

### Preoperative Diagnostics and Preparations

All patients with incisional hernias underwent a preoperative CT scan. In cases where a W3 defect was present, preconditioning with botulinum toxin was performed 4 weeks prior to surgery. The indication for a TAR procedure was determined preoperatively based on the Carbonell Index, which considers the ratio of the rectus muscle diameter to the hernia defect diameter. For primary hernias, an ultrasound examination of the abdominal wall was conducted.

### Postoperative Follow-Up

All patients who underwent robotic surgery were scheduled for a follow-up appointment in our outpatient clinic approximately 6 weeks postoperatively. During this visit, patients received a comprehensive assessment of their general wellbeing, as well as a physical and an ultrasound examination. Special attention was paid to retro-muscular seromas and hematomas, as well as local wound complications.

## Results

A total of 160 patients underwent ventral hernia repair using the lateral approach eTEP technique. The median age was 62 years (range: 26–87), with 97 male patients (60.63%) and 63 female patients (39.37%). The median ASA score was 2 (range: 1–3). Patients had a median height of 1.73 m (range: 1.52 m–2.06 m) and a median weight of 89.5 kg (range: 50 kg–145 kg), resulting in a median BMI of 30 (range: 22–45) (see Table 1).

**TABLE 1:** General patient characteristics.

		all	n = 160	eTEP	n = 117	eTAR	n = 43
Age (years)	Median/Range	62.00	(23–87)	63.00	(26–87)	60.00	(23–85)
Male	Number/Percentage	97	60.63%	75	64.10%	22	51.16%
Female	Number/Percentage	63	39.38%	42	35.90%	21	48.84%
ASA	Median/Range	2	(1–3)	2	(1–3)	2	(1–3)
Height (m)	Median/Range	1.73	(1.52–2.06)	1.73	(1.52–2.06)	1.72	(1.54–1.87)
Weight (kg)	Median/Range	89.5	(50–145)	90	(50–145)	82	(53–128)
BMI (kg/m2)	Median/Range	30	(20–45)	30	(20–45)	29	(20–42)

The median operative time was 143 min (range: 53 min–495 min). The median length of hospital stay was 3 days (range: 2–16). Among the cases, 111 (69.38%) were incisional hernias and 49 (30.63%) were primary ventral hernias. The median mesh size was 540 cm^2^ (range: 225–1,350), with a median hernia defect size of 25 cm^2^ (range: 2.25–375). This results in a median defect-to-mesh ratio (MDR) of 21 (range: 2.33–150). 9 simultaneous inguinal hernias were treated with additional mesh during the operation. In 5 cases it was a unilateral inguinal hernia, in 2 cases it was a bilateral finding. 139 patients presented with a medial hernia defect (86.98%), seven patients (4.14%) presented with lateral hernias, and 14 (8.88%) presented with combined hernias. A total 43 cases required TAR for complete fascial closure (30 unilateral TAR and 13 bilateral TAR) out of which 22 patients suffered a medial hernia defect. All 21 patients with lateral or combined hernias needed TAR to close the defect and ensure a sufficient mesh overlap. Complete fascial closure was obtained in all cases (see Table 2). All TAR procedures were preoperatively indicated based on the ratio of rectus muscle diameter to hernia defect diameter. An unplanned intraoperative extension of the procedure to include a TAR was not necessary.

**TABLE 2:** Intra- and postoperative parameters.

		all	n = 160	eTEP	n = 117	eTAR	n = 43
Operating Time (min)	Median/Range	143	(53–495)	125	(68–351)	238	(53–495)
Length of Stay (days)	Median/Range	3	(2–16)	3	(2–9)	4	(2–16)
Incisional Hernias	Number/Percentage	111	69.38%	69	58.97%	42	97.67%
Primary Hernias	Number/Percentage	49	30.63%	48	41.03%	1	2.33%
Mesh Size (ventral hernia)	Median/Range	540	(225–1,350)	510	(260–880)	750.00	(225–1,350)
Mesh Size (all hernias)	Median/Range	560	(225–1,392)	510	(260–984)	756.00	(225–1,392)
Defect Size	Median/Range	25	(2.25–375)	16	(2.25–200)	120	(4.5–375)
MDR	Median/Range	21	(2.33–150)	30	(3.06–150)	5.92	(2.33–80)
Medial Hernia	Number/Percentage	139	86.98%	117	100.00%	22	51.16%
Lateral Hernia	Number/Percentage	7	4.14%	0	0.00%	7	16.28%
Combined Hernia	Number/Percentage	14	8.88%	0	0.00%	14	32.56%
Complete Defect Closure	Number/Percentage	160	100.00%	117	100.00%	43	100.00%

There was one intraoperative complication involving a patient with a previous skin transplantation after open abdomen, where multiple serosal tears occurred during complex adhesiolysis. This necessitated a segmental resection of the small intestine with subsequent anastomosis. Despite the complexity of the case, the patient’s postoperative course was without further complications.

In two additional cases, both involving large L4 hernias after partial nephrectomy, an intraoperative conversion to open preperitoneal repair was required after completion of the robotic transversus abdominis release (TAR). The conversion was necessary due to extensive scarring caused by the use of a hemostyptic cellulose agent during the previous operation in the retroperitoneum. The postoperative course of both patients was uneventful (see Table 3).

**TABLE 3:** Intra- and postoperative complications including conversion rates.

		all	n = 160	eTEP	n = 117	eTAR	n = 43
Complications	Number/Percentage	11	6.88%	7	5.98%	4	9.30%
Intraoperative	Number/Percentage	1	0.63%	0	0%	1	2.32%
Postoperative	Number/Percentage	10	6.25%	7	5.98%	3	6.98%
Conversions	Number/Percentage	2	1.25%	0	0.00%	2	4.65%
Clavien Dindo 1	Number/Percentage	6	3.75%	5	4.27%	1	2.33%
Clavien Dindo 2	Number/Percentage	2	1.25%	1	0.85%	1	2.33%
Clavien Dindo 3a	Number/Percentage	0	0.00%	0	0.00%	0	0.00%
Clavien Dindo 3b	Number/Percentage	2	1.25%	1	0.85%	1	2.33%
Clavien Dindo 4	Number/Percentage	0	0.00%	0	0.00%	0	0.00%
Clavien Dindo 5	Number/Percentage	0	0.00%	0	0.00%	0	0.00%

The postoperative complication rate was 6.25% (10 patients), with 1.72% (2 patients) requiring reoperation. In five patients, a seroma was identified during sonographic follow-up, occurring four times in the retromuscular mesh space and once in the former hernia sac; none of these cases required surgical intervention. A superficial wound healing disorder around a trocar incision was treated conservatively.

Additionally, one patient experienced an acute episode of chronic pancreatitis during hospitalization, while another developed postoperative pneumonia. One patient developed a hematoma, which was evacuated via laparoscopy Another patient experienced temporary neuropraxia of the femoral nerve due to a positioning injury, which consequently led to an ankle fracture requiring surgical stabilization.

## Discussion

*“No disease of the human body, belonging to the province of the surgeon, requires in its treatment, a better combination of accurate, anatomical knowledge with surgical skill than hernia in all its varieties”* – Sir Aston Pestley Cooper [17].

Though bold and perhaps applicable across all surgical disciplines, this quote by renowned surgeon and anatomist Sir Astley Paston Cooper from 1804 underscores the enduring challenge and anatomical complexity of hernia surgery, which probably remained unchanged to this day.

The integration of robotics into hernia surgery has introduced new possibilities for the implementation of minimally invasive techniques with extraperitoneal mesh placement, while also allowing for new anatomical considerations. Enhanced visualization and precision offered by robotic systems have enabled surgeons to explore novel approaches to accessing and repairing hernias.

In 2021, Baur et al [18] investigated two of these robotic adaptations involving transperitoneal access in a large group of 118 patients with ventral and incisional hernias in Switzerland: rv-TAPP (“robotic ventral transabdominal preperitoneal plasty”) with preperitoneal mesh implantation and r-TARUP with retrorectus mesh implantation (r-Rives). This study demonstrated that robotic surgery combines the advantages of minimally invasive procedures (low complication rate) with the advantages of open procedures (morphological reconstruction) and enables consistent extraperitoneal mesh placement.

In 2018, Belyansky first described a robotic adaptation of the eTEP technique. As previously mentioned, unlike transabdominal procedures, this approach opts for a total extraperitoneal access route, initially dissecting the non-preoperated layers of the abdominal wall, far from the hernia site. In a feasibility study involving 37 patients with ventral, incisional, lateral, or parastomal hernias, Belyansky demonstrated the safety and feasibility of robot-assisted hernia surgery utilizing an extraperitoneal approach [11].

Given the absence of a control group for this study, we have compared our results with the available data in the literature in order to contextualize our results.

In 2020, Morrell et al. [19] reported on technical standardisation and their anatomical considerations in robot-assisted eTEP repair of ventral hernias and described 10 key steps for safe and reproducible repair. In 2021, Kudsi and Gokcal [20] reported on the short-term results after using a lateral eTEP approach with and without transversus abdominis release (TAR) in 52 patients. Quezada et al. released postoperative data from a case series involving 66 patients in 2022 [21].

In the study conducted by Morell et al., no intraoperative complications were observed among the 22 patients included. However, one postoperative seroma was reported, resulting in a surgical complication rate of 4.5%. Reoperation was not required in any of the three studies analyzed. In another case series by Belyansky et al., the authors documented no intraoperative complications, but two postoperative seromas necessitating interventional drainage. This corresponds to a surgical complication rate of 5.4% among the 37 patients included in this study. Kudsi and Gokcal reported a series of 52 patients. Similarly, their results showed no intraoperative complications or conversions. Nonetheless, two seromas and one hematoma following a fall were identified, resulting in a postoperative complication rate of 5.8%. Quezada et. al reported one recurrence (1.5%) and 10 surgical site occurrences (15%) including 6 seromas, 2 hematomas and 2 surgical site infections. Four patients out of their series required reinterventions (6%).

Over a span of more than 4 years, we encountered similar results and complication rates. Regarding the reoperations in our series, one involved an open reduction and internal fixation for an ankle fracture caused by a fall, which resulted from neuropraxia of the femoral nerve due to positioning-related overextension. Notably, in our series, there were no cases of suture rupture of the posterior rectus sheath or peritoneum, which can lead to intraparenchymal hernias and is considered one of the primary risks associated with new minimally invasive procedures involving extraperitoneal mesh placement. Additionally, four patients in our cohort developed significant retromuscular seromas postoperatively, none of which required intervention.

One possible reason for this low number of seroma complications is the consistent postoperative application of an abdominal binder in combination with retromuscular drain as recommended by Mazzola Poli de Figueiredo et al. [22] in their review article published in 2023. Furthermore, in a systematic review published in 2023 by Marcolin et al. [23], there appears to be evidence of a significant reduction in postoperative seromas through drain placement. In accordance to this finding a drain was placed above the mesh at the end of every operation regardless of intraoperative fluid accumulation. In comparison, the complication rate of 6.25% is consistent with the data published in current literature.

Moreover, the robotic systems offer an excellent modality for a totally extraperitoneal adaptation and safe implementation of a posterior component separation as described by Novitsky [24].

Although there are currently no specific publications highlighting the benefits of the extraperitoneal approach in hernia surgery, we have observed significant advantages in the fact that the robotic system can be introduced into a space where no prior surgeries have been performed, thereby avoiding adhesions. This is particularly valuable in cases of incisional hernia surgery, where intraperitoneal adhesions are ought to be expected.

Given the fact, that a large portion of ventral hernia operations involve incisional hernias, the choice of access and trocar placement is paramount for the success of the operation. In this context and in contrast to transperitoneal techniques, there appears to be a particular advantage, in that the robot, can be docked in the retrorectus space without requiring major adhesiolysis.

Unlike transperitoneal techniques, intraperitoneal adhesiolysis is rarely required and typically limited to a small area after opening the hernial sac. However, based on our experience, the use of robotic systems offers a distinct advantage in performing adhesiolysis. This benefit has been reflected in recent literature, where robotic surgery appears to be associated with a lower rate of bowel injuries compared to laparoscopic control groups [25, 26].

On the other hand, the possibility of unnoticed transperitoneal damage to the intestine poses a significant risk during surgery. Prakhar [27] et al. reported two cases of occult bowel injuries that required surgical revision on the second postoperative day. A possible cause for such injuries could be the uncontrolled use of monopolar energy, potentially leading to damage from leakage currents in areas of thinned peritoneum, as commonly found in scar tissue.

Additionally, by minimizing intraperitoneal adhesiolysis, there could be potential for a further reduction in the rate of bowel injuries, similar to the outcomes observed in minimally invasive inguinal hernia surgery. In a study reported by Felix et al. in 1995 [28], the authors compared large numbers of cases involving TEP and TAPP surgeries. Their results showed a significantly lower number of bowel injuries in patients treated with the TEP technique. The same result was found in the study by Tamme [29] in 2003, where none of the over 5000 TEP patients experienced a bowel injury.

During the initial stages of our robotic surgery program, transperitoneal procedures were preferred, in accordance with the recommended training pathway of the European Hernia Society (EHS), due to the complexity of extraperitoneal techniques. Currently, the extraperitoneal eTEP technique has become the standard approach in our hospital, even for complex hernias. It is also routinely used for large W3 hernias requiring bilateral TAR, as intraperitoneal adhesions are often present in this situation and access problems are to be expected.

To minimize suture tension, our hospital utilizes a technique similar to the MILOS (Mini/Less Open Sublay) method, in which the posterior rectus sheath is left open, and only the peritoneum is repaired to preserve the integrity of the abdominal wall.

Furthermore, trocar positioning offers a distinct advantage compared to transperitoneal techniques, where trocars are typically placed more laterally along the anterior axillary line during lateral docking. In extraperitoneal procedures, trocars are positioned along the midclavicular line, providing more space between the ribcage and iliac crest. This allows for greater distance between the individual incisions, resulting in greater range of motion of the individual robotic arms, thereby reducing the risk of collisions with the patient or the operating table. Additionally, this approach eliminates the need for placing the patient in an overextended position, as often required in bottom-up or top-down techniques.

In contrast to transperitoneal procedures (TARUP) trocar insertion sites are extraperitoneal and covered by mesh, herein reducing the risk of subsequent trocar hernias.

We encountered a limitation related to the width of the rectus muscle, which ideally should measure at least 5-6 cm. This requirement arises from the fact that the distance from the remote center of the robotic trocars, positioned at the fascial entry point, to the instrument tip measures 6 cm. This technical constraint, inherent to the robotic system in use, must be acknowledged as it directly influences the maneuverability and effectiveness of the instruments within the confined retrorectus space.

For the dissection of the retrorectus space at the beginning of the operation, a section of native tissue is required at the lateral edge of the rectus sheath on one side of the body. Extensive scarring or pararectus access routes that extend far laterally thus pose limitations to the method. This constraint is similarly applicable in cases of reoperations where previously implanted meshes occupy the entire retrorectus space, as these meshes may obstruct access and hinder effective dissection.

In case of early accidental pneumoperitoneum, the procedure may potentially increase in complexity. With sufficient experience in robotic or endoscopic extraperitoneal surgeries, this typically does not pose a significant issue. At the beginning of the learning curve however, it may necessitate a conversion to a transperitoneal or even open approach. Therefore, robotic eTEP should not be considered a “beginner’s operation” or a training procedure [30] for robotic surgery but rather be trained gradually through hands-on courses and proctoring in order to shorten the learning curve, similarly to its endoscopic counterpart as demonstrated by Korneffel et al [31].

## Conclusion

The “lateral approach” of robotic eTEP offers a safe surgical method for treating ventral hernias with minimal invasive techniques and mesh augmentation in the retro-muscular space. Given appropriate expertise even in complex cases of large W3 hernias or lateral hernias, a complete fascial closure can be achieved by performing TAR. The major limitations of the method are confined to cases that have been previously operated on in the retro-muscular space or present scars in the access areas. Compared to transperitoneal procedures, there appear to be specific advantages with regard to reduced need for adhesiolysis and improved trocar positioning in extraperitoneal approaches. However, further comparative studies are necessary to better understand the relative benefits and outcomes of these approaches. This would provide a more comprehensive assessment of their clinical advantages, particularly in terms of patient safety, recovery, and long-term efficacy.

## Data Availability

The original contributions presented in the study are included in the article/supplementary material, further inquiries can be directed to the corresponding authors.
